# In utero hyperthermia in late gestation derails dairy calf early-life mammary development

**DOI:** 10.1093/jas/skac186

**Published:** 2022-10-07

**Authors:** Bethany M Dado-Senn, Sena L Field, Brittney D Davidson, Geoffrey E Dahl, Jimena Laporta

**Affiliations:** Department of Animal and Dairy Sciences, University of Wisconsin-Madison, Madison, WI 53706, USA; Department of Animal and Dairy Sciences, University of Wisconsin-Madison, Madison, WI 53706, USA; Department of Animal and Dairy Sciences, University of Wisconsin-Madison, Madison, WI 53706, USA; Department of Animal Sciences, University of Florida, Gainesville, FL 32611, USA; Department of Animal and Dairy Sciences, University of Wisconsin-Madison, Madison, WI 53706, USA

**Keywords:** epithelium, heat stress, histology, mammary, prenatal, proliferation

## Abstract

Prenatal hyperthermia has immediate and long-term consequences on dairy cattle growth, immunity, and productivity. While changes in the molecular architecture are reported in the mature mammary gland (**MG**), any influence on early-life mammary development is unknown. Herein, we characterize the impact of late-gestation in utero heat stress on heifer mammary gross and cellular morphology at early-life developmental stages (i.e., birth and weaning). During summer, pregnant dams were exposed to environmental heat stress (shade of a free-stall barn) or offered active cooling (shade, fans, and water soakers) for 54 ± 5 d before parturition (avg. temperature–humidity index = 79). Heifer calves born to these dams were either in utero heat-stressed (**IU-HT**; *n* = 36) or in utero cooled (**IU-CL**; *n* = 37) and were managed as a single cohort thereafter. A subset of heifers was euthanized at birth (**d0**; *n* = 8/treatment; 4.6 ± 2.3 h after birth) and after weaning (**d63**; *n* = 8/treatment; 63.0 ± 1.5 d) to harvest the whole MG. An ultrasound of rear mammary parenchyma (**MPAR**) was taken prior to d63 and correlated to harvested MPAR cross-sectional area and weight. Portions of mammary fat pad (**MFP**) and MPAR were preserved for compositional and histological analysis, including ductal structure number and cross-sectional area, connective tissue area, and adipocyte number and cross-sectional area. Cellular proliferation in MPAR was assessed via Ki-67 immunohistochemistry. Relative to IU-CL heifers, the MGs of IU-HT heifers were shorter in length at d0 and d63 (*P* ≤ 0.02). There were moderate correlations between d63 ultrasound and harvest measures. The IU-HT heifers had reduced MG and MFP mass at d0 and d63 (*P* ≤ 0.05), whereas MPAR mass was reduced only at d0 (*P* = 0.01). IU-HT heifers had greater MPAR protein and DNA content at d63 (*P* ≤ 0.04), but there were no MFP compositional differences (*P* ≥ 0.12). At d0, IU-HT heifers had fewer MPAR ductal structures (*P* ≤ 0.06), but there were no differences at d63. Yet, MPAR luminal and total ductal structure cross-sectional areas of IU-HT heifers were reduced at both d0 and d63 (*P* ≤ 0.01). The MFP adipocytes of IU-HT heifers were smaller at d0 (*P* ≤ 0.01), but differences were not detected at d63. The IU-HT heifers had diminished MPAR total, stromal, and epithelial cellular proliferation at both d0 and d63 (*P* < 0.01). Prenatal hyperthermia derails dairy calf early-life mammary development with potential carry-over consequences on future synthetic capacity.

## Teaser

Dairy heifers exposed to in utero heat stress have smaller mammary glands and fat pads at birth and weaning relative to body weight. The portion of these glands responsible for future milk synthesis has reduced ductal development and cellular proliferation, which could impact productive capacity later in life.

## Introduction

Late-gestation heat stress has severe, long-lasting consequences on dairy cattle dam and offspring phenotype and productivity. Dairy cattle exposed to intrauterine hyperthermia in late gestation are born lighter and earlier, have impaired passive immune transfer, and have lower survivability in the herd relative to cattle born under thermoneutral conditions, despite similar management postnatally (Dado-Senn et al., 2020; Laporta et al., 2020). Indeed, in utero heat-stressed heifers that survive to lactation produce less milk over multiple lactations (Monteiro et al., 2016; Laporta et al., 2020). This reduction in milk yield is partially attributed to profound alterations in lactating mammary tissue architecture and an unfavorable methylation landscape. Specifically, the mammary glands (**MG**s) of lactating heifers exposed to in utero heat stress have a greater proportion of connective tissue and smaller alveoli, the milk synthesizing structures of the gland (Skibiel et al., 2018a). Furthermore, prenatally heat-stressed heifers have an altered epigenome, with differently methylated genes related to transcription, translation, gene regulation, cell differentiation, and cell development (Skibiel et al., 2018b). Nevertheless, these data were obtained from mature, lactating cows 2 yr after the initial prenatal heat stress insult. Additional work is needed to characterize the impact of late-gestation hyperthermia on mammary form and function across the entire lifetime of the dairy cow, starting in early life to elucidate the underlying mechanisms leading to the reduction in milk yields.

Mammary growth and development are initiated in utero, with the formation of the mammary band and bud in early gestation and exponential mammary growth in late gestation (Bauman and Currie, 1980; Akers, 2017). At birth, the MG consists of a large fat pad (i.e., stromal tissue) and a relatively smaller parenchyma (i.e., future synthetic tissue) with rudimental ductal branching. After birth, there is an increase in fat pad mass and parenchyma ductal elongation (Akers, 2002). This mammary growth has been historically classified as isometric from birth through the weaning (2 to 3 mo of age) and allometric until puberty attainment around 10 mo, though this dogma has recently been challenged to suggest an earlier initiation of the allometric phase (Capuco and Akers, 2010). Since the foundation for mammary function is laid in utero and in the first few months of life, exposure to stressors prenatally or early postnatally could greatly affect immediate and long-term mammary morphology and synthetic capacity (Knight and Sorensen, 2001; Akers, 2002). For instance, preweaned calves fed a limited plane of nutrition have reduced early-life total mammary, fat pad, and parenchyma mass; blunted parenchyma cellular proliferation, and even lower milk yield upon maturity (Brown et al., 2005; Davis Rincker et al., 2011; Geiger et al., 2016, 2017).

Our lab recently demonstrated that late-gestation maternal heat stress, and consequent in utero hyperthermia, reduced offspring organ mass in early life, including the MG (Dado-Senn et al., 2021). However, additional investigation into mammary morphology and tissue architecture was warranted. The objective herein was to determine the impact of late-gestation hyperthermia on dairy heifer mammary growth and development at birth and weaning through invasive and noninvasive techniques. We assessed mammary gross morphology, mammary fat pad (**MFP**) and parenchyma (**MPAR**) composition and microstructure, and MPAR cellular proliferation after organ harvest at 0 and 63 d of age. We hypothesized that heifers exposed to heat stress in utero would have smaller MGs, decreased MFP and MPAR mass and cross-sectional area, reduced MPAR ductal structure number and cross-sectional area, and diminished MPAR cellular proliferation, DNA, and protein content, relative to in utero thermoneutral counterparts.

## Materials and Methods

All procedures were approved by the University of Florida Institutional Animal Care and Use Committee (protocol #201910599). This study was performed from August to December 2020 in northern Florida. Pregnant dams were managed on a commercial dairy farm, and calves were reared at a University of Florida campus facility from birth to weaning.

### Dam treatment and calf management

A detailed description of dam and calf treatment and management can be found in Dado-Senn et al. (2021). Briefly, multiparous pregnant Holstein dams were dried off at 54 ± 5 d before parturition and simultaneously enrolled into heat-stress (**HT**; *n* = 41) or cooling (**CL**; *n* = 41) treatments. The HT dams had access to the shade of the free-stall barn, whereas CL dams had shade, fans, and water soakers. Continuous recording of pen temperature–humidity index (**THI**) averaged 79 across the treatment period, indicating cows were above the THI thresholds for dry period heat stress (Ouellet et al., 2021). In addition, dam respiration rate (53.5 vs. 77.3 ± 0.6 breaths per min for CL vs. HT dams, respectively) and skin temperature (34.1 vs. 36.0 ± 0.05 °C) were different between maternal treatments (*P* < 0.001; Dado-Senn et al., 2021), confirming the cooling treatment efficacy in restoring thermoneutrality. Heifers born to these dams were either in utero heat-stressed (**IU-HT**; *n* = 36) or in utero cooled (**IU-CL**; *n* = 37) during late gestation.

Heifers were managed identically as a single cohort from birth to weaning, fed 0.87 kg dry matter (DM) per d milk replacer (12% solids, Supplementary Table S1) over two feeding periods with milk replacer weaning starting at 49 d and ending at 56 d of age. Starter grain concentrate (Supplementary Table S1) and water were offered ad libitum from birth. Dry matter intake was recorded daily and averaged weekly, and body weight (**BW**) was recorded at 0, 28, 56, and 63 d of age (Dado-Senn et al., 2021). Briefly, the entire population of IU-HT heifers weighed 4 to 6 kg less than IU-CL heifers from birth (34.4 vs. 39.2 ± 0.9 kg for IU-HT vs. IU-CL, respectively; *P* < 0.001) to weaning (78.9 vs. 84.9 ± 1.0 kg; *P* < 0.001). However, subsets of heifers euthanized for further mammary assessment did not have a difference in BW at the time of euthanasia (Table 1). There was no difference between treatments for milk replacer intake, but IU-HT calves consumed less starter concentrate from 6 to 9 wk of age relative to IU-CL calves (*P* ≤ 0.08). The total dry matter intake was reduced in IU-HT calves by roughly 0.13 kg/d across this period.

**Table 1.** Mammary gland gross morphology and composition^1^

	d0				d63			
	IU-CL	IU-HT	SEM	*P*-value	IU-CL	IU-HT	SEM	*P*-value
Body weight (BW, kg)	37.99	36.29	1.01	0.25	83.44	80.42	1.89	0.28
Mammary gland (MG) weight, composition								
Untrimmed MG, g	145.66	125.34	6.74	0.05	400.00	332.50	22.41	0.05
Untrimmed MG, g/kg BW	3.84	3.49	0.22	0.29	4.80	4.14	0.25	0.09
Trimmed MG, g	119.90	102.48	5.57	0.05	247.61	193.59	15.50	0.03
Trimmed MG, g/kg BW	3.16	2.66	0.13	0.02	2.97	2.41	0.18	0.04
Mammary fat pad (MFP, g)	95.21	79.44	2.71	0.001	221.03	179.10	11.80	0.03
MFP, g/kg BW	2.52	2.20	0.08	0.01	2.65	2.23	0.13	0.04
MFP, g/g MG	0.79	0.76	0.02	0.32	0.93	0.93	0.01	0.90
MFP protein, mg/g MFP	—	—	—	—	14.49	12.55	0.88	0.15
MFP total protein, g	—	—	—	—	2.61	2.24	0.23	0.27
MFP DNA, mg/g MFP	—	—	—	—	0.31	0.31	0.01	0.70
MFP total DNA, mg	—	—	—	—	61.83	56.25	2.37	0.12
Mammary parenchyma (MPAR, g)	0.31	0.14	0.04	0.01	7.84	9.39	0.99	0.29
MPAR, g/kg BW	0.008	0.004	0.001	0.01	0.10	0.12	0.01	0.28
MPAR, g/g MG	0.003	0.001	0.0003	0.01	0.035	0.050	0.004	0.03
MPAR protein, mg/g MPAR	—	—	—	—	36.11	38.91	0.75	0.02
MPAR total protein, g	—	—	—	—	0.29	0.37	0.04	0.19
MPAR DNA, mg/g MPAR	—	—	—	—	1.40	1.82	0.13	0.03
MPAR total DNA, mg	—	—	—	—	10.86	20.25	2.86	0.04
MG measures								
MG length, cm	15.21	11.85	0.73	0.01	25.33	21.38	1.00	0.02
MG width, cm	10.44	9.63	0.54	0.31	13.14	13.05	0.63	0.92
Distance fore-to-rear teats, mm	35.00	31.670	1.280	0.09	48.07	45.69	1.73	0.35
Distance between fore teats, mm	55.29	48.63	2.08	0.04	73.00	69.50	2.42	0.33
Distance between rear teats, mm	35.25	35.63	1.82	0.89	48.86	49.50	2.32	0.85
Fore teat length, mm	14.88	14.38	1.03	0.74	19.29	19.06	1.31	0.91
Rear teat length, mm	13.66	13.00	0.98	0.64	18.07	17.94	1.23	0.94

Dairy heifers were euthanized at birth (d0) and after weaning (d63) after exposure to either in utero heat stress (IU-HT) or cooling (IU-CL, *n* = 8 per treatment and timepoint) for the last 54 ± 5 d of gestation to harvest their mammary glands. Gross morphology assessments include mammary weight (untrimmed and trimmed of excess skin), length, width, teat length, and distance. Mammary parenchyma and fat pad DNA and protein composition were also assessed.

### MG gross measures

**Figure 1.**
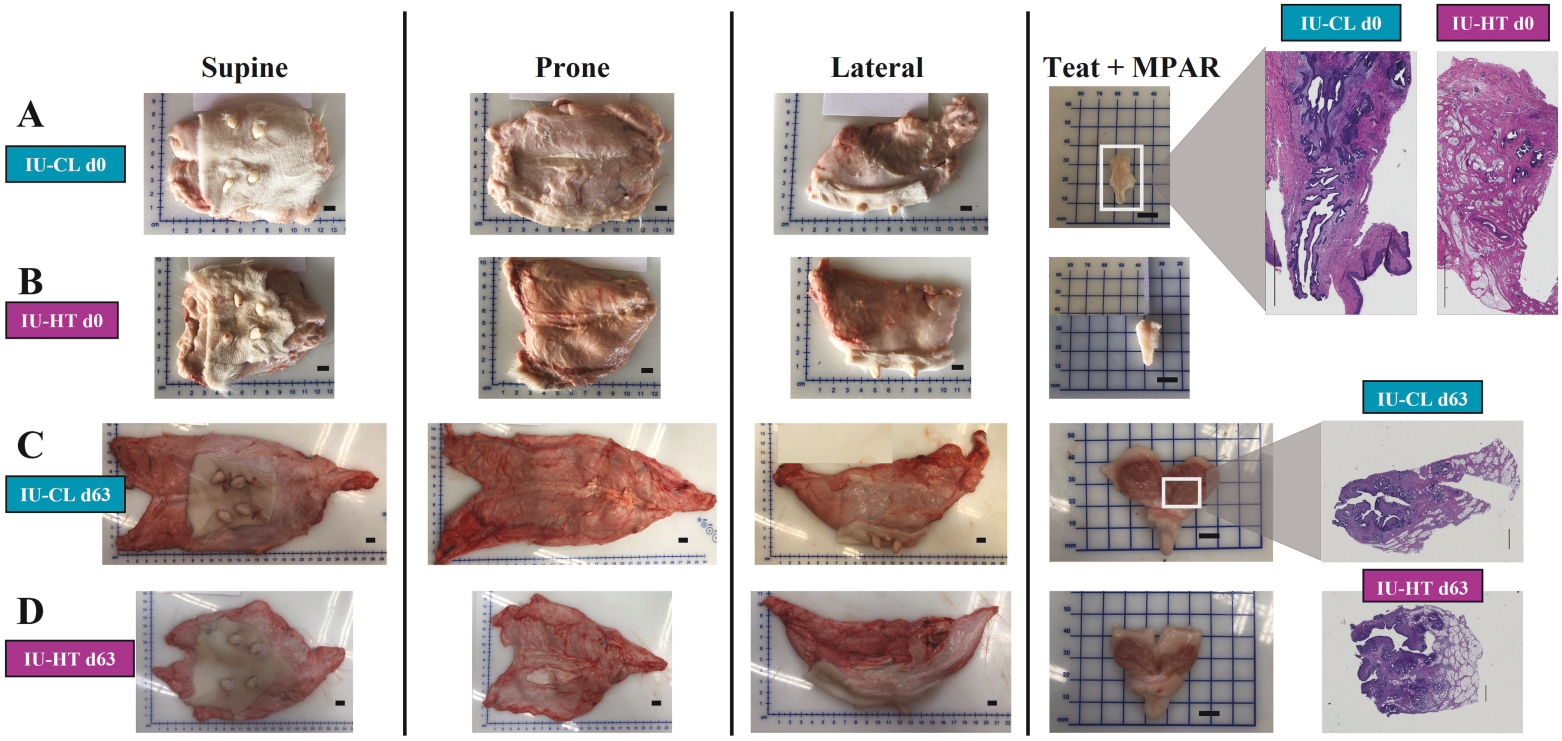
Pictographic representation of bovine mammary glands. Mammary glands were harvested at birth (d0, A, B) and after weaning (d63, C, D) after exposure to in utero heat stress (IU-HT) or cooling (IU-CL, *n* = 8 per treatment and timepoint) during the last 54 ± 5 d of gestation. From left to right, pictures represent the trimmed (i.e., excess skin and lymph node removed) supine, prone, and lateral MG, bisected rear teat, and histological H&E-stained representation of mammary parenchyma. The MG images were captured via a digital camera (scale bar = 1 cm). The tissue photomicrograph was captured by stitching 4× images using the Keyence BZ-X800 microscope and software (scale bar = 2,000 μm). Abbreviations: H&E, hematoxylin and eosin; MG, mammary gland; MPAR, mammary parenchyma.

A subset of heifers was euthanized at birth via captive bolt and exsanguination (**d0**; *n* = 8 per treatment; 4.6 ± 2.3 h after birth). Another subset of calves was euthanized after weaning (**d63**; *n* = 8 per treatment; 63.0 ± 1.5 d of age). The MG was removed within 10 min after complete exsanguination, weighed to capture untrimmed MG weights, and trimmed of excess skin and the supramammary lymph nodes to obtain trimmed MG weights (Figure 1). Weights were assessed as gross weight and on a per kilogram of BW basis. The MG length (cranial–caudal distance) and width, front and rear teat lengths, distance from front to rear teats, distance between front teats, and distance between rear teats were measured using an electronic digital micrometer caliper (Vinca, DCLA-0405).

### MG tissue collection

At d0, the MG was bisected into the right and left portions along the median suspensory ligament. The right rear quarter was dissected to collect MPAR and MFP for histological analysis. The MPAR present at birth was visible as a thin brown line of tissue (Akers, 2002; Meyer et al., 2006), so for proper orientation, the entire MPAR within the teat was preserved (Figure 1A and B). The two tissue portions were preserved according to Field et al. (2021) until paraffin embedding. The remaining quarters were dissected to accrue MPAR and MFP portions (~10 to 100 mg) that were stored in RNAlater (ThermoFisher, Invitrogen; #AM7020, Grand Island, NY) or snap-frozen in liquid nitrogen for DNA and protein isolation. The remaining MFP was weighed to quantify the total MFP weight, and the MPAR collected from the remaining three quarters was divided by three and multiplied by four to obtain the total MPAR weight. Tissue weight was assessed as gross weight and as a ratio to both kilogram of BW and gram of MG.

**Figure 2.**
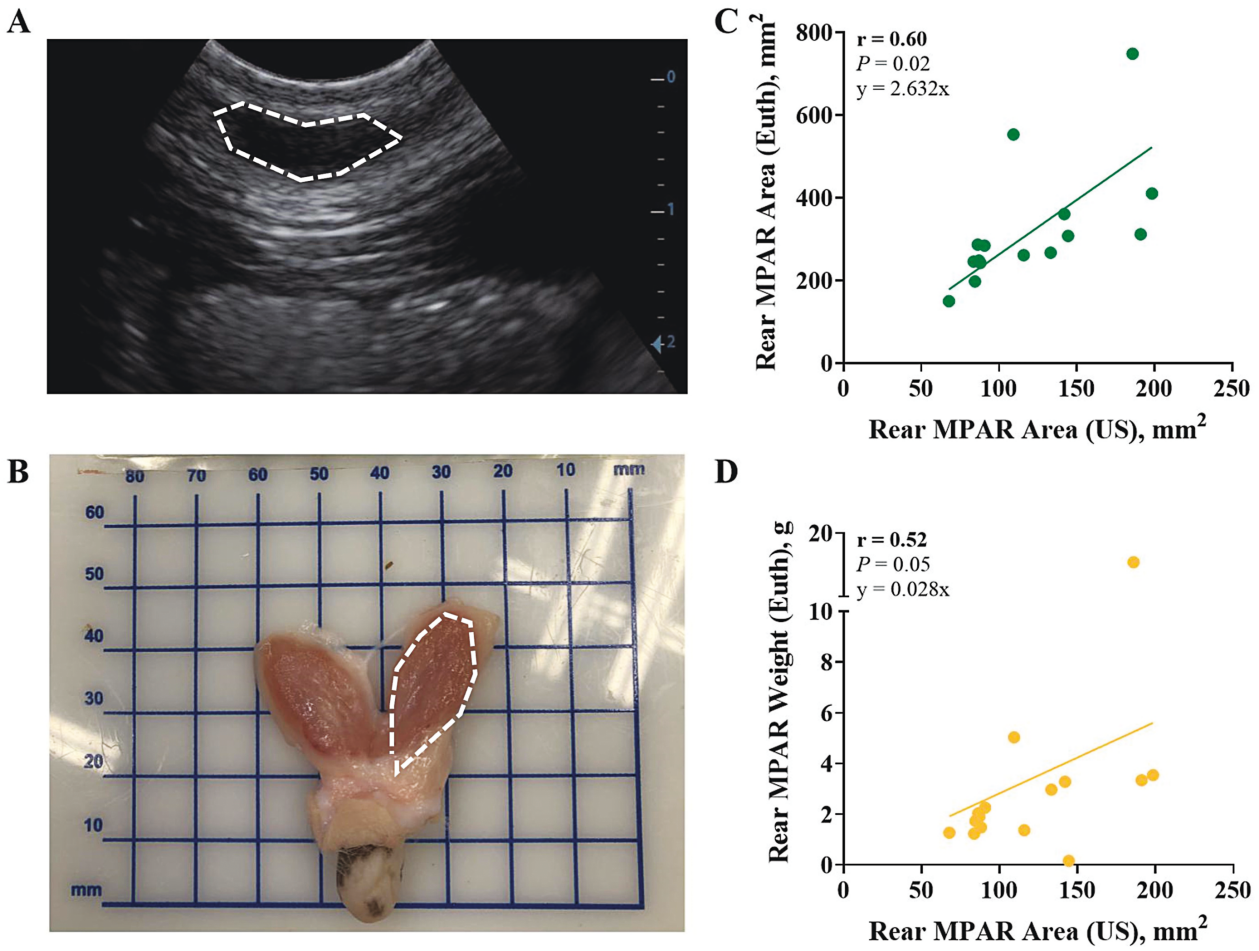
Correlations between ultrasound (A) rear mammary gland surface area and cross sectional surface area (B, C) or weight (D) at 63 d of age. Rear MPAR surface area was assessed noninvasively at d63 using ultrasonography (A) and quantified using ImageJ (*n* = 16). The cross-sectional surface area was assessed invasively through euthanasia (B) by harvesting the mammary gland and dissecting the rear MPAR. Digital images were taken and quantified by using ImageJ (*n* = 15), and weight was recorded. Abbreviation: MPAR, mammary parenchyma; US, ultrasound; Euth, Euthanasia.

The d63 tissue collection protocol followed that of the birth euthanasia with adjustments following Geiger et al. (2016). After trimming and collecting gross measures, the MG was bisected along the median suspensory ligament, and the right half was divided into front and rear quarters. The right rear quarter was selected for histological analysis of MPAR and MFP. The MPAR region within the teat was bisected to the top of the teat (Figure 1C and D) and fixed according to Field et al. (2021). After fixation, these tissues were further subsampled as (1) parenchyma proximal to the teat (i.e., proximal MPAR; **MPARP**) and (2) the outermost parenchyma and the surrounding MFP (i.e., distal MPAR; **MPARD**). A separate portion of MFP from near the MPAR region was subsampled and fixed for histological evaluation. Cross-sectional MPAR surface area was captured digitally from the right rear quarter after bisection by area tracing in ImageJ (Figure 2B).

The left half of the MG was wrapped in aluminum foil and stored at −80 °C until further processing. The frozen half of MG was trimmed, and the MPAR and MFP were dissected. The left-portioned MG, MPAR (front and rear), and MFP were weighed and multiplied by two to determine the total trimmed MG, MPAR, and MFP weight. Samples were pulverized and then stored at −80 °C for the quantification of DNA and protein.

### MPAR ultrasonography

The day prior to d63 euthanasia, an ultrasound was used to visually assess the MPAR area according to Esselburn et al. (2015) and Vang et al. (2021). From above the rear teats, four ultrasound images per teat per heifer (*n* = 8 per treatment) were captured. MPAR area was detected on the upper portion of the image as a hypoechoic ultrasound element (Figure 2A; Vang et al., 2021). The MPAR structures were traced in ImageJ, and measurements from the left and right rear teat were averaged to quantify the rear MPAR area. The MPAR area captured through ultrasound (i.e., noninvasive) was used to estimate correlations with rear MPAR weight and surface area captured after organ harvest (i.e., invasive; Figure 2B). Harvested MPAR surface area was assessed in ImageJ from digital images collected at euthanasia. The MPAR image from one calf was not captured, so correlations were conducted from *n* = 16 datapoints for MPAR weight and *n* = 15 datapoints for MPAR surface area.

### Cellular histology and immunohistochemistry

The MPAR and MFP from d0 and the MPARP, MPARD, and MFP from d63 were paraffin-embedded, sectioned at 5 μm, and affixed to slides. The d0 MPAR and d63 MPARP were stained with hematoxylin and eosin (**H&E**) and Masson’s trichrome according to Dado-Senn et al. (2019). The d0 MFP and d63 MFP were stained with Masson’s trichrome only. These stained slides were used to identify, analyze, and quantify mammary tissue microstructure, connective and epithelial tissue area, and adipocyte characteristics. To assess cellular proliferation, Ki67 (Mouse anti-Human Ki67, DAKO #M7240, clone MIB-1) immunohistochemistry was performed on d0 MPAR and d63 MPARD according to Field et al. (2021). Proliferating cells stained brown (positive) and non-proliferating cells stained blue (negative). Positive and negative controls can be found in Supplementary Figure S1.

### Quantification of histological sections

Photomicrographs of MPAR and MFP were captured using the Keyence BZ-X800 microscope and analyzed in ImageJ or Keyence BZ-X800 Analyzer hybrid cell counter. Capture exposure and illumination as well as analyzer software target area thresholds can be found in Supplementary Table S2.

For H&E-stained MPAR sections, four photomicrographs were captured and averaged per section at 4× magnification to quantify MPAR ductal structure number and cross-sectional area. Ductal structures were considered any epithelial structure in the MPAR, characterized as either luminal (i.e., containing a lumen) or non-luminal (i.e., containing no lumen; an epithelial “cluster”). Ductal structures were counted using the Point Picker plugin (Thévenaz 2010) and traced for cross-sectional area quantification in ImageJ. Area measurements were calibrated to image size and magnification (i.e., pixels to μm).

For the Masson’s trichrome-stained MPAR sections, 4 (d0) or 10 (d63) photomicrographs per section were captured at 10× magnification and used to quantify epithelial (purple) and connective tissue area (blue) using the BZ-X800 software hybrid cell count tool. Intraparenchymal adipocyte cell count and area (individual and total) were also quantified using ImageJ tracing.

Masson’s trichrome-stained MFP was captured as four photomicrographs per section at 20× magnification. Images were processed to reverse negative/positive to convert the connective tissue stain surrounding adipocytes to a black color and all other tissue stains to white. The hybrid cell count tool partitioned individual adipocytes in each photomicrograph, quantifying extraparenchymal adipocyte cell count and cross-sectional area.

The Ki67 immune-stained MPAR sections were captured as four photomicrographs per section at 40× magnification. In each capture, every cell was counted and defined as positive (brown) or negative (blue) using the hybrid cell count tool. Cells were classified as either epithelial (i.e., encompassing the ductal structures) or stromal (i.e., surrounding cells such as fibroblasts and adipocytes) by visual tracing before employing the hybrid cell count.

### Protein and DNA content

The pulverized tissue from the frozen left MG half was used to determine MPAR and MFP protein and DNA content per milligram of tissue and total tissue content. Protocols followed that of Daniels et al. (2009a) and Geiger et al. (2016). Protein content was determined using the bicinchoninic acid Protein Assay Kit (ThermoScientific, Prod# 23227) according to the manufacturer’s instructions. Absorbency was measured using a spectrophotometer plate reader (Spectra Max 190, A&K Biosource Inc.; 562 nm wavelength). The intra-assay coefficient of variation (CV) was 3.4%. The DNA content was determined via fluorimetry according to Daniels et al. (2009a) with modifications. A standard curve developed from calf thymus (1 mg/mL, Sigma Aldrich #D4522) was plated in a 96-well plate, and then 2 μL supernatant and 200 μL assay solution were plated in triplicate. The assay solution consisted of 100 μL of 1 mg/mL Hoechst H33258 stain (Sigma Aldrich #94403) + 10 mL of 2 M NaCl, 10mM Tris, and 10 mM Na_2_EDTA (Ethylenediaminetetraacetic acid disodium salt) + 90 mL of ddH_2_O (double distilled H_2_O) at a pH of 7.4. Samples were measured using a fluorimeter microplate reader (Synergy H1 Hybrid Reader, BioTek; fluorescence = 465 nm, excitation = 355 nm). Intra-assay CV was 4.6%. Protein and DNA composition are reported as concentration (milligram per gram of tissue) and total.

### Statistical analysis

Data were analyzed in SAS (v. 9.4 SAS Institute, Cary, NC). All residuals were tested for normality, and the first-order autoregressive covariance structure (AR-1) was used as the covariance structure. Data including mammary measures, histology, and composition were analyzed as single timepoint variables where d0 and d63 were analyzed separately as two-sample *t*-tests with the main effect of in utero treatment. Cell proliferation was analyzed PROC GLIMMIX as a generalized linear mixed model with a binomial distribution and logit link function. Results are expressed as the proportion of positive cells to total cells (positive + negative, %) per section total and for each cell type (i.e., epithelial and stromal). Pearson correlations between ultrasound rear MPAR area and harvested rear MPAR area and weight were calculated using the CORR procedure. Data are presented as least square means (LSM) ± standard error (SE) unless otherwise stated. Significance was declared at *P* ≤ 0.05 and tendency at 0.10 ≥ *P* > 0.05.

## Results

### MG morphology and composition

Mammary gross morphology and composition outcomes are summarized in Table 1. In utero heat-stressed heifers had smaller untrimmed and trimmed MGs at d0 and d63 relative to IU-CL counterparts (*P* ≤ 0.05), and this difference remained significant when adjusted for BW except for d0 untrimmed MG. Similarly, the MFP weighed less in IU-HT heifers at d0 and d63, both gross and adjusted for BW (*P* ≤ 0.04). Yet, there were no differences between treatments for the protein or DNA content in MFP on d63 (*P* > 0.12).

There was a 3-fold reduction in MPAR weight (both gross and adjusted to BW) in IU-HT heifers at birth (*P* = 0.01), but no statistical differences were detected for MPAR weight on d63 relative to IU-CL heifers (*P* ≥ 0.28). Still, IU-HT MPAR had significantly greater protein and DNA concentration relative to IU-CL heifers at d63 (*P* ≤ 0.03). When assessing MPAR weight as adjusted to MG weight (i.e., proportion of MPAR in MG), IU-HT heifers had less MPAR per gram of MG at d0 but significantly more MPAR per gram of MG at d63 relative to IU-CL heifers.

The MG of IU-HT heifers were shorter in length at d0 with a tendency for decreased distance from fore-to-rear teats and significantly reduced distance between fore teats, compared with IU-CL heifers (*P* ≤ 0.09). Only the reduction in mammary length remained in IU-HT heifers at d63 (*P* = 0.02). There were no other significant differences in MG width, teat distances, or teat lengths on d63 (*P* ≥ 0.33).

### MPAR cross-sectional area

There was no difference between treatments for MPAR surface cross-sectional area measured either by ultrasound imaging (125.8 vs. 130.3 ± 18.8 mm^2^ for IU-CL and IU-HT respectively; *P* = 0.89) or by image tracing after euthanasia tissue harvest (250.4 vs. 296.1 ± 25.4 mm^2^; *P* > 0.23) at approximately 63d of age. Regardless of in utero treatments, there was a moderate, positive (*r* = 0.60, *P* = 0.02) correlation between heifer rear MPAR area as measured by ultrasound (i.e., noninvasive) and rear MPAR area assessed after euthanasia (i.e., invasive; Figure 2C). The correlation between ultrasound rear MPAR area and harvested rear MPAR weight was also moderate and positive (Figure 2D; *r* = 0.52, *P* = 0.05).

### MPAR and fat pad microstructure

**Figure 3.**
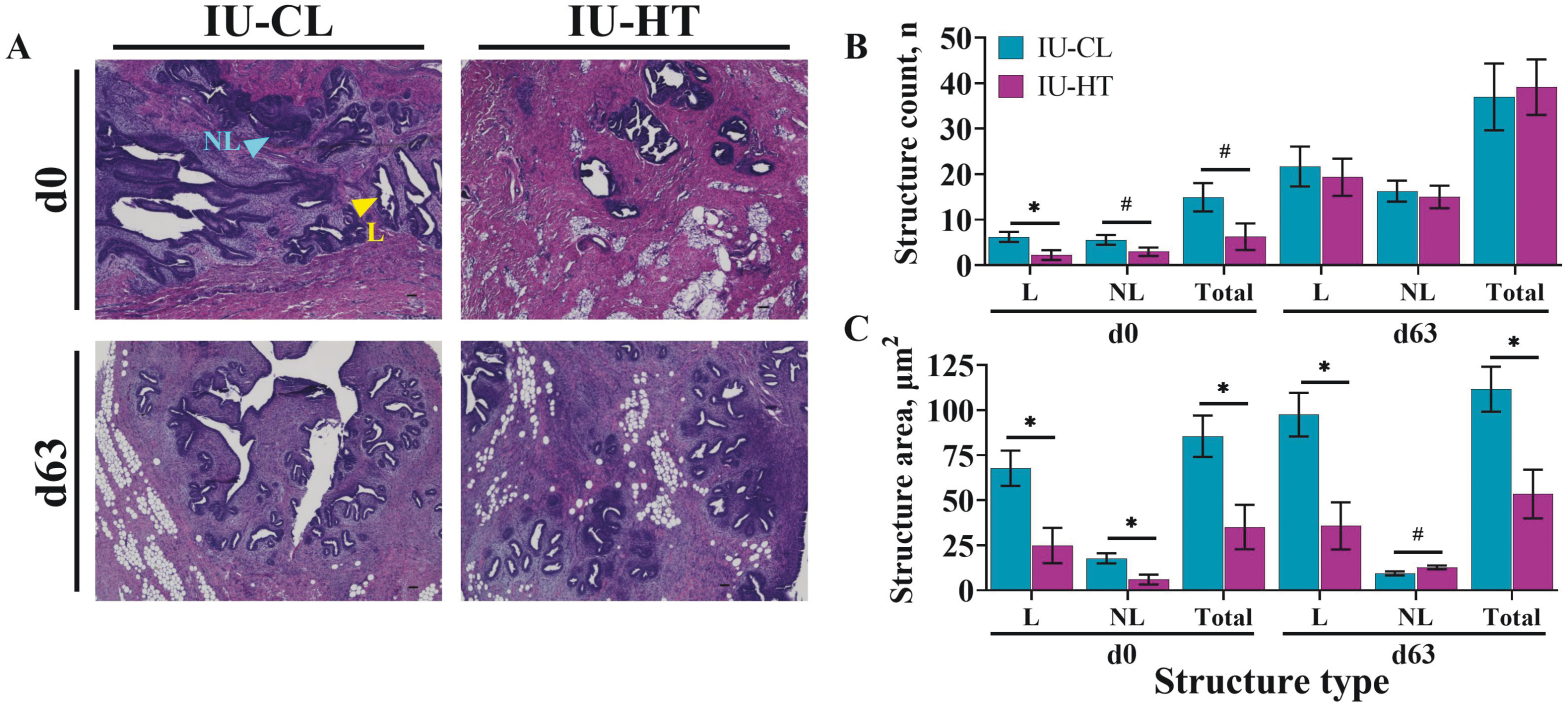
Histological evaluation of early-life mammary gland parenchyma microstructure via H&E-stained photomicrographs. MPAR of dairy heifers was harvested at birth (d0) and after weaning (d63) after prenatal exposure to in utero heat stress (IU-HT) or cooling (IU-CL, *n* = 8 per treatment and timepoint) during the last 54 ± 5 d of gestation. MPAR H&E photomicrographs (A) were captured at 4× (scale bar = 500 μm); L depicts a luminal and NL depicts non-luminal epithelial ductal structures. Parenchymal luminal, non-luminal, and total ductal structure count (B) and cross-sectional area (C) were quantified between treatments at d0 and d63. Asterisk (*) indicates *P* ≤ 0.05 and # indicates 0.10 ≥ *P* > 0.05. Abbreviations: H&E, hematoxylin and eosin; MPAR, mammary parenchyma.

At d0, IU-HT heifers had fewer MPAR luminal ductal structures (*P* = 0.02) and tended to have fewer non-luminal ductal structures (*P* = 0.09) relative to IU-CL counterparts. Consequently, IU-HT heifers tended to have less total ductal structure number (Figure 3A and B; 15 vs. 6 ± 3 ductal structures for IU-CL vs. IU-HT respectively; *P* = 0.06). On d63, MPAR ductal structure count was no longer different between treatments (Figure 3A and B; *P* ≥ 0.69). The IU-HT heifers had significantly reduced luminal and total MPAR ductal structure cross-sectional area compared with IU-CL heifers at d0 (Figure 3A and C; 85.5 vs. 35.1 ± 11.9 μm^2^; *P* = 0.01) and d63 (Figure 3A and C; 111.7 vs. 53.4 ± 13.0 μm^2^; *P* = 0.01).

**Figure 4.**
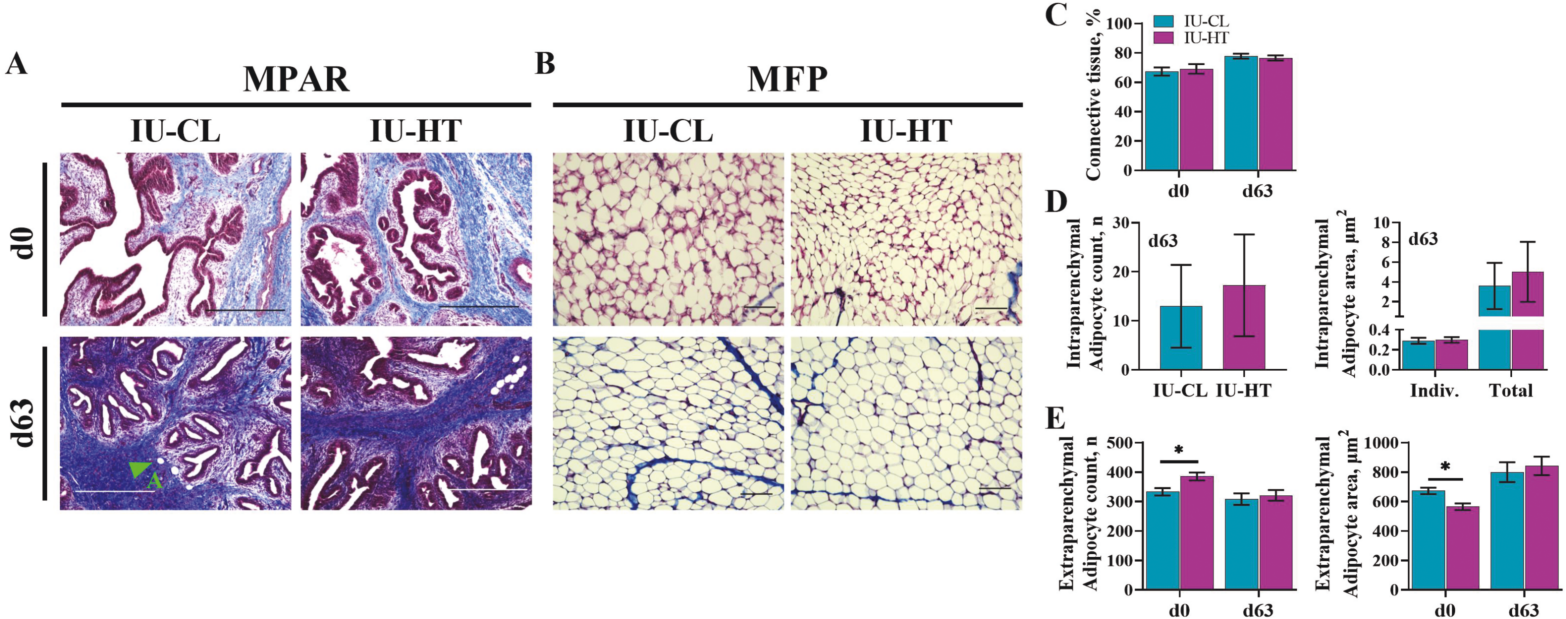
Histological evaluation of early-life parenchyma and fat pad microstructure via Masson’s trichrome-stained photomicrographs. Mammary parenchyma and fat pad of dairy heifers harvested at birth (d0) and after weaning (d63) after prenatal exposure to in utero heat stress (IU-HT) or cooling (IU-CL, *n* = 8 per treatment and timepoint) during the last 54 ± 5 d of gestation. MPAR photomicrographs (A) were captured at 10× (scale bar = 500 μm; arrow = adipocyte) to determine MPAR connective tissue area between in utero treatments at d0 and d63 (C) as well as intraparenchymal adipocyte count and cross-sectional area (D) at d63. MFP photomicrographs (B) were captured at 20× (scale bar = 100 μm) to determine extraparenchymal adipocyte count and cross-sectional area (E) between in utero treatments at d0 and d63. Asterisk (*) indicates *P* ≤ 0.05. Abbreviations: MFP, mammary fat pad; MPAR, mammary parenchyma.

There were no differences between in utero treatments for MPAR connective tissue proportions at d0 or d63 (Figure 4A and C; *P* ≥ 0.62). The intraparenchymal adipocyte cross-sectional area and adipocyte count were highly variable and did not differ between treatments (Figure 4D, *P* ≥ 0.71). The MFP of IU-HT heifers at d0 had greater extraparenchymal adipocyte number with reduced cross-sectional area relative to IU-CL heifers (Figure 4B and E; *P* ≤ 0.01). There were no differences between treatments for MFP adipocyte count or area at d63 (Figure 4B and E; *P* ≥ 0.64).

### MPAR cellular proliferation

**Figure 5.**
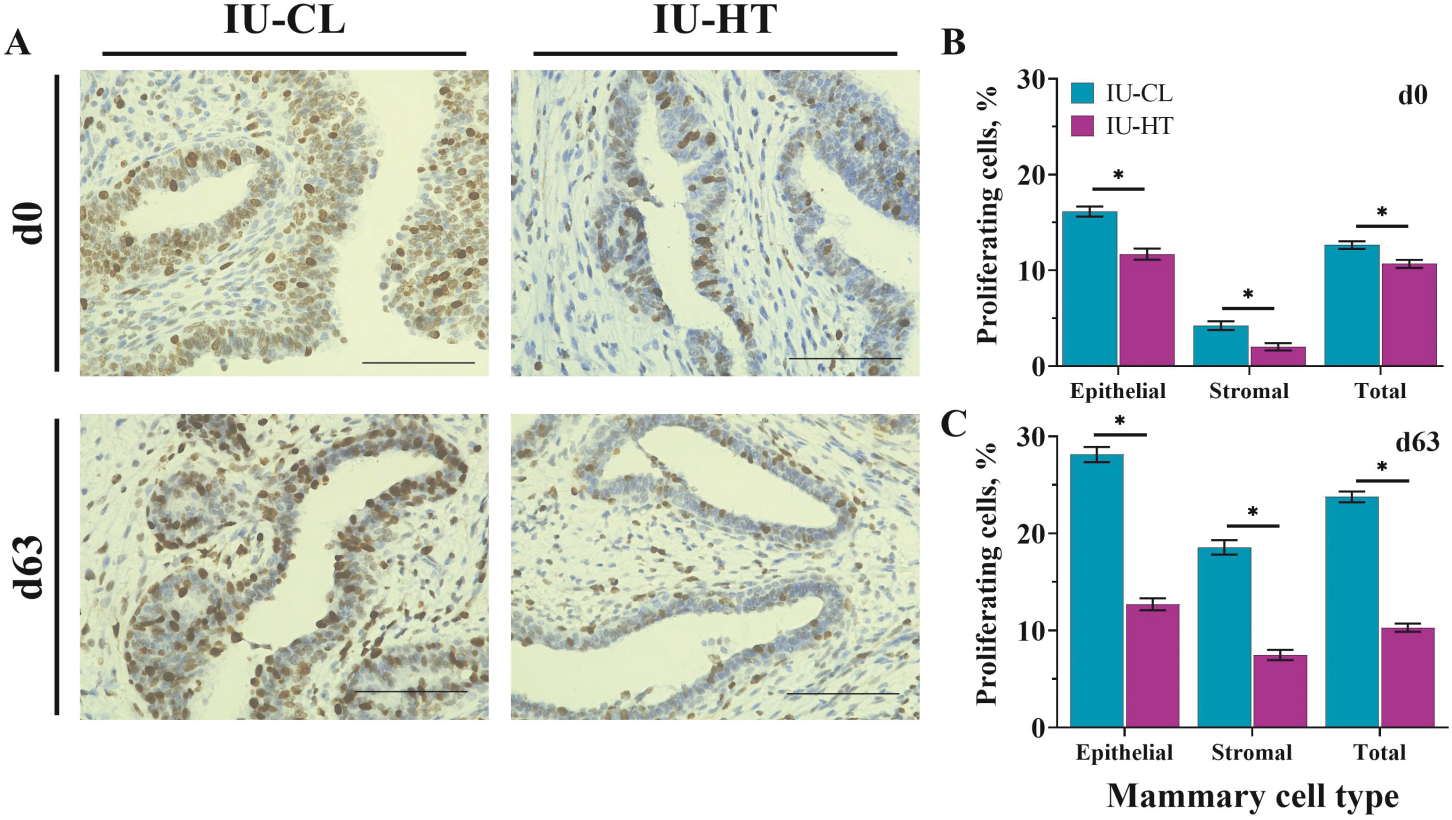
Immunohistochemistry evaluation for cellular proliferation in the early-life mammary gland. Ki67-stained photomicrographs (A) of mammary parenchyma in dairy heifers at birth (d0) and after weaning (d63) after exposure to either in utero heat stress (IU-HT) or in utero cooling (IU-CL, *n* = 8 per treatment and timepoint) during the last 54 ± 5 d of gestation. The images were captured at 40× (scale bar = 100 μm). Cellular proliferation was assessed as a ratio of positive cells (dark brown) to total cells and determined for epithelial, stromal, and total (epithelial + stromal) mammary cell types at d0 (B) and d63 (C). A photomicrograph of the positive and negative controls along with relative proliferation rates can be found in Supplementary Figure S1. Asterisk (*) indicates *P* ≤ 0.05.

Heifers exposed to in utero hyperthermia had reduced total MPAR cellular proliferation at both d0 (Figure 5A and B; 12.64 vs. 10.68 ± 0.41%, *P* = 0.006) and d63 (Figure 5A and C; 23.76 vs. 10.29 ± 0.48%, *P* < 0.001) relative to IU-CL heifers. This reduction in cell proliferation was evident in both the epithelial and stromal portions of the MPAR (Figure 5).

## Discussion

Late-gestation in utero heat stress severely limits milk yield in dairy cattle over multiple lactations, even years after the initial insult (Monteiro et al., 2016; Laporta et al., 2020). While we have characterized the impact of this stressor on alterations in mature mammary histology and methylation profile (Skibiel et al., 2018a, 2018b), additional investigation into early-life windows of mammary development is warranted to fully elucidate the lifelong consequences of intrauterine heat stress on offspring mammary morphology and microstructure. Herein, we measured mammary gross morphology and mammary fat pad and parenchyma composition, tissue microstructure, and cellular proliferation at birth and after weaning in heifers exposed to late-gestation prenatal hyperthermia.

Heifers exposed to late-gestation in utero heat stress had reduced trimmed MG and MFP mass at birth and weaning, relative to in utero cooled heifers. Reduced overall birth and weaning BWs are a hallmark of late-gestation in utero heat stress (Tao et al., 2019). This was also reported in the present study (Dado-Senn et al., 2021), though the subsets of heifers euthanized did not have a difference in BW at the time of euthanasia. Regardless of the lack of BW difference, the difference in mammary size remains when accounting for BW. Reduction in mammary mass, including the fat pad, has previously been reported at weaning in dairy heifers fed a limited plane of nutrition (Brown et al., 2005; Daniels et al., 2009a; Geiger et al., 2016). However, these studies likely detected differences due to direct reduction in nutrient availability preweaning, while causes for a lighter MG herein could be multi-factorial. We posit the decrease in mammary mass triggered by in utero hyperthermia could be attributed to impaired fetal nutrient availability and altered blood flow from reduced placental development and function (Tao et al., 2019; Casarotto et al., 2021) or a reduction in postnatal nutrient availability due to the inherent reduction in grain intake (Dado-Senn et al., 2021). Another plausible explanation may be the alterations in methylation profile contributing to dysregulation in key genes leading to reduced cellular proliferation and organ growth (Skibiel et al., 2018a)

The difference in total mammary mass is likely primarily driven by fat pad accumulation in IU-CL heifers relative to IU-HT. Further analysis of the MFP indicates that there is no difference between treatments for fat pad protein and DNA content or adipocyte characteristics at weaning; however, the fat pad of IU-HT heifers has smaller yet more numerous adipocytes at birth. It is possible that in utero heat stress might limit adipocyte hypertrophy in utero or shortly after birth, contributing to the smaller adipocytes and subsequent reduced MFP mass (Neville et al., 1998; Zaboli et al., 2017). However, it seems that at least individual adipocyte size and number recovered by weaning. The MFP arises from the mesodermal layer of mammary development, into which mammary epithelial cells of ectodermal origin will proliferate and invade as further development occurs. Thus, the composition and quantity of MFP influence the resulting degree of epithelial invagination, ductal morphogenesis, and overall gland structure (Sheffield, 1988; Hovey et al., 1999; Hovey and Aimo, 2010). Though accelerated growth of the MFP during the postweaning allometric growth phase has been shown to inhibit parenchyma development (Sejrsen et al., 1982; Capuco et al., 1995), expansion of the MFP preweaning, as observed in the IU-CL heifers, seems to have a little-to-no negative impact on synthetic parenchymal growth (Thorn et al., 2008; Daniels et al., 2009a). Indeed, enhancing MFP growth appears to promote the genetic crosstalk between the MFP and MPAR to support MPAR development (Vailati-Riboni et al., 2018).

Interestingly, while IU-HT heifers sustained a lighter MFP across early life, their MPAR mass was smaller only at birth with no gross mass difference detected at weaning. Parenchymal mass is often reported in studies assessing the impact of preweaning nutrient plane on mammary development, though outcomes are contradictory. Some studies find no difference in MPAR mass at weaning (Meyer et al., 2006; Daniels et al., 2009a), whereas others report a robust increase in MPAR under elevated planes of nutrition (Brown et al., 2005; Geiger et al., 2016). Notably, this research model is imposed postnatally so MPAR mass at birth is not impacted. Indeed, the only study that harvested the udder around birth did not quantify MPAR mass (Meyer et al., 2006). Therefore, it is difficult to determine if less MPAR at birth could have any short- or long-term effect on mammary development and function.

Accompanying the smaller MPAR of IU-HT heifers were severe reductions in MPAR ductal growth and cellular proliferation at birth, both of which suggest the potential for a less-developed MG. Interestingly, these alterations were not transient and were also observed after weaning. The complexity of mammary ductal development in prepubertal heifers was previously estimated by assessing the number of epithelial structures, particularly those containing a lumen, as well as the amount of tissue area occupied by epithelium (Daniels et al., 2009b). Those researchers found that the number of MPAR luminal structures increased linearly with BW, but neither age nor BW influenced the percentage of epithelium in MPAR tissue (Daniels et al., 2009b). Similarly, heifers exposed to in utero hyperthermia had fewer ductal structures at birth that appeared to be less developed at both birth and weaning, as indicated by a significantly reduced luminal ductal structure area. The development of mammary secretory tissue (i.e., alveoli) during gestation is dependent on the foundation of a ductal network in the peripubertal period. It is possible that derailing the development of the ductal epithelium early in life (i.e., prenatally) impairs future secretory tissue development and milk production at maturity (Swanson, 1960; Little and Kay, 1979; Sejrsen et al., 2000).

Decreased MPAR cellular proliferation is likely a key driver of the reduced MPAR mass at birth and diminished complexity of ductal development in IU-HT heifers. Heifers exposed to in utero heat stress had a substantial reduction in cellular proliferation in MPARD epithelium and stroma at both birth and weaning relative to in utero cooled counterparts. This MPARD is the outermost parenchyma, closest to the surrounding MFP. Reduced proliferation at this location could affect the degree of epithelial growth and invagination into the fat pad during mammary development. Remarkably, the relative rates of proliferation of IU-HT heifer MPAR remained around 10% at both birth and after weaning, while the rate of proliferation of IU-CL heifer MPAR essentially doubled after weaning (12% vs. 24%). The 10% MPAR proliferation rate in IU-HT heifers is similar to the rate in MPARD of weaned heifers fed at a restricted plane of nutrition reported by Geiger et al. (2017). Interestingly, the rate of proliferation in the terminal ductal units of enhanced-fed heifers in that study was 26%, comparable to the proliferation rate of the IU-CL heifers at weaning (Geiger et al., 2017). However, proliferation by ductal location was not assessed in our study, and only the MPARD was quantified.

The magnitude of heifer parenchymal development is important, as the number of mammary epithelial cells at maturity is considered one of the primary factors limiting milk production (Tucker, 1981). The impaired cellular proliferation of IU-HT heifers in early life could impact mammary epithelial cell number or activity and milk potential in maturity. We have shown that in utero heat-stressed heifers have an increased proportion of connective tissue, accompanied by decreased luminal-alveolar area, reduced mammary epithelial cell number, and lower milk production in their first lactation (Monteiro et al., 2016; Skibiel et al., 2018a). Notably, this mammary phenotype is observed 2 yr after the initial prenatal insult, indicating a persistent effect on limiting MG growth potential.

Notably, the lack of MPAR mass difference at weaning, despite a difference at birth, could be indicative of compensatory growth of IU-HT MPAR between 0 and 63 d of age. This supposition is supported by the elevated MPAR protein and DNA concentration and total DNA content of IU-HT heifers after weaning, also demonstrated in enhanced-fed heifers (Brown et al., 2005). However, these results are contradictory to the above-mentioned reduction in cellular proliferation found in IU-HT heifers at weaning. We initially posited that the cause for this contradiction could be a rise in intraparenchymal adiposity of weaned IU-HT heifers influencing MPAR mass. However, there were no differences detected between treatments for intraparenchymal adipocyte count or area in the MPAR of weaned heifers. Therefore, our current hypothesis is that IU-HT heifers have a shifted window of MPAR growth than that of IU-CL heifers such that accelerated cellular proliferation was not detected in IU-HT heifers at 63 d of age. This supposition warrants further investigation at different stages of life or a deeper dive into the hormonal profile of the MFP and MPAR around weaning. For instance, MPAR growth between birth and weaning could be less sensitive to steroid and peptide hormone profile relative to MPAR growth in late gestation. Pregnant dams exposed to late gestation heat stress have altered estrone-sulfate and thyroid hormone profiles which could influence the offspring’s MPAR development at birth (Collier et al., 1982), leading to compensatory gain in IU-HT heifer MPAR in a relatively quiescent period of parenchymal development.

Since the results discussed above require an invasive, endpoint assessment of mammary development, other noninvasive mammary measures could be employed to assess the impact of intrauterine hyperthermia on mammary growth more feasibly. Ultrasounds of rear MPAR after weaning detected no difference between treatments for mammary surface area, and they were moderately correlated with the rear MPAR surface area and weight as measured after organ harvest (which were not different between treatments). This technology has recently been implemented in dairy heifer research to assess general MG development (Nishimura et al., 2010) or impacts of dietary manipulation on MPAR growth and development (Albino et al., 2015; Esselburn et al., 2015; Vang et al., 2021). Its application could serve as a proxy to determine MPAR size but not relative cellular proliferation or development at weaning (Nishimura et al., 2010). However, the moderate association between harvested and ultrasound-imaged MPAR documented here and in a similar study (Esselburn et al., 2015) is a limitation to the use of this technology.

Noninvasive mammary and teat measures were also assessed in the present study. The MGs of IU-HT heifers were shorter in length at both birth and weaning, and there were similar reductions in teat distance at birth. Interestingly, calves under a restricted diet also have reduced mammary lengths relative to those under a more enhanced diet (Geiger et al., 2016), though differences in teat distance were not detected. While it is notable that these reductions in length align with the decrease in mammary mass after exposure to in utero heat stress, these morphological measures on their own have little value in predicting the long-term synthetic capacity of the MG (Akers, 2002).

In order to assess the molecular characteristics of the MG, endpoint organ harvests were necessary and prevented us from following animals longitudinally. Therefore, we cannot directly link the reductions in mammary mass or MPAR cellular proliferation to impaired mature milk production. However, it is well established that early-life stressors can have long-term implications on livestock productive outcomes (Van Eetvelde and Opsomer, 2017). Similarly, implementing intensive planes of nutrition in preweaned calves improves weaning-age body mass and milk yields in the first lactation (Davis Rincker et al., 2011). Those authors suggest that an intensive preweaning diet with subsequent improvement in first-lactation milk production could be related to elevated mammary fat pad and parenchyma mass, DNA concentration, and development, as reported in a similar study (Brown et al., 2005). Likewise, we suggest that perturbations in mammary mass and parenchymal development that begin at or even before birth could contribute to the mammary development and milk synthesis impairment consistently documented in mature in utero heat-stressed cows (Monteiro et al., 2016; Skibiel et al., 2018a; Laporta et al., 2020; Ouellet et al., 2020). A subset of heifers from disparate in utero treatments is currently being evaluated for mammary development in later stages that precede lactation, including puberty and gestation.

## Conclusion

Dairy heifers exposed to late-gestation in utero heat stress have lighter and smaller MGs at birth and approximately 1 wk after weaning relative to their in utero thermoneutral counterparts. This difference appears to be driven by a large reduction in MFP mass. The MPAR of in utero heat-stressed heifers is smaller at birth with diminished ductal development and reduced cellular proliferation. These differences were observed at birth and maintained after weaning. The impaired ductal complexity and epithelial cell growth in early life may limit mammary epithelial development and synthetic capacity, contributing to the altered mammary microstructure and reduced milk yield reported in in utero heat-stressed heifers at maturity.

## Supplementary Material

skac186_suppl_Supplementary_Material

## Abbreviations

BW: body weight
H&E: hematoxylin and eosin
L: luminal ductal structure
MFP: mammary fat pad
MG: mammary gland
MPAR: mammary parenchyma
MPARD: distal MPAR
MPARP: proximal MPAR
NL: non-luminal ductal structure
THI: temperature–humidity index
US: ultrasonography
