# Supplementary material for: In utero hyperthermia in late gestation derails dairy calf early-life mammary development
Source: J Anim Sci. 2022 Oct 7;100(10):skac186. doi: 10.1093/jas/skac186 (PMC9541282; doi:10.1093/jas/skac186)
Supplement: skac186_suppl_Supplementary_Material [file skac186_suppl_supplementary_material.docx]

Supplementary Materials –***In utero* hyperthermia in late-gestation derails dairy calf early-life mammary development,** Dado-Senn et al. 2022

**Supplementary Table S1.** Ration formulation and nutrient content of far-off and close-up TMR fed to heat-stressed and cooled dry cows, as well as milk replacer and starter grain fed to heifers.

|  | Pregnant cow diet^1^ | |  | Calf diet | |
| --- | --- | --- | --- | --- | --- |
| Item | Far-off | Close-up |  | Milk replacer^2^ | Starter grain^3^ |
| **Ingredient, % of DM** |  |  |  |  |  |
| Corn silage | 27.36 | 45.92 |  | - | - |
| Concentrate | 3.28 | 23.71 |  | - | - |
| Canola meal | 0.84 | 4.38 |  | - | - |
| Sorghum silage | 21.35 | - |  | - | - |
| Close-up weighback | 28.47 | - |  | - | - |
| Bermuda grass green chop | 8.89 | - |  | - | - |
| Gin trash | 9.80 | - |  | - | - |
| Grass silage | - | 13.03 |  | - | - |
| Cotton hulls | - | 12.95 |  | - | - |
| **Nutrient Content (DM basis)** |  |  |  |  |  |
| Moisture, % | 69.47 | 56.70 |  | 3.80 | 11.20 |
| Dry Matter, % | 30.53 | 43.28 |  | 96.20 | 88.80 |
| CP, % | 14.00 | 15.27 |  | 28.90 | 23.73 |
| Fat, % | 3.63 | 3.36 |  | 20.61 | 2.99 |
| ADF, % | 33.18 | 30.00 |  | - | 14.95 |
| aNDF, %^4^ | 48.43 | 43.63 |  | - | 28.25 |
| NFC, %^4^ | 29.53 | 32.33 |  | - | 39.90 |
| Lignin, % | 6.51 | 6.06 |  | - | 3.49 |
| Ethanol soluble CHO, % | 2.90 | 3.70 |  | - | - |
| Starch, % | 13.87 | 16.93 |  | - | - |
| Ash, % | 7.51 | 8.19 |  | 6.47 | 6.76 |
| Ca, % | 0.72 | 0.89 |  | 0.71 | 1.00 |
| P, % | 0.31 | 0.33 |  | 0.68 | 0.57 |
| Mg, % | 0.26 | 0.35 |  | 0.11 | 0.28 |
| K, % | 1.43 | 1.48 |  | 1.42 | 1.52 |
| Na, % | - | - |  | 0.61 | 0.26 |
| NE_L_ (Mcal/kg)^5^ | 1.38 | 1.41 |  | - | - |
| NE_G_ (Mcal/kg)^5^ | 0.38 | 0.40 |  | 3.40 | 0.55 |
| ME (Mcal/kg)^5^ | 2.28 | 2.34 |  | 4.92 | 1.26 |

^1^ Far-off diet was fed from 222 to 243 d gestation (~56 d prepartum) and close-up diet was fed from 244 d gestation to calving (~277 d gestation).

^2^ UF Special 28/15 Bova DFB Medicated, Southeast Milk, Okeechobee, FL

^3^ Ampli-Calf Starter 20 Warm Weather, Purina Animal Nutrition LLC, Shoreview, MN

^4^ NDFa = ash-free NDF; NFC = non-fiber carbohydrate, NFC = DM – (ash + CP + ether extract + NDF – NDF insoluble CP).

^5^ Calculated using the NRC (2001) according to the chemical composition of the diet and adjusted to cow or calf.

**Supplementary Table S2.** Properties used to capture and analyze histological images of calf mammary proximal or distal parenchyma (MPAR, MPARP, MPARD) and fat pad (MFP) at birth (d0) and after weaning (d63).

|  |  |  | **Microscope images** | | |  | **Hybrid cell count** | | | |
| --- | --- | --- | --- | --- | --- | --- | --- | --- | --- | --- |
| Stain | Tissue | Euthanasia | Magnification | Brightness (Exposure) | Transmitted light |  | Image processing | Threshold, target area | Threshold, 1^st^ extraction | Threshold, 2^nd^ extraction |
| H&E | MPAR | d0 | 4X | 1/300 | 50 |  | . | . | . | . |
|  | MPARP | d63 | 4X | 1/300 | 50 |  | . | . | . | . |
| Masson’s | MPAR | d0 | 10X | 1/100 | 50 |  | . | 235 | 40, 50, 60, 70 | . |
|  | MPARP | d63 | 10X | 1/100 | 50 |  | . | 235 | 35, 40, 65, 90 | . |
|  | MFP | d0 | 20X | 1/100 | 50 |  | Reverse neg/pos | 250 | 46 | . |
|  | MFP | d63 | 20X | 1/100 | 50 |  | Reverse neg/pos | 250 | 60 | . |
| Ki67 | MPAR | d0 | 40X | 1/30 | 50 |  | Haze reduction | 250 | 156 | 95 |
|  | MPARD | d63 | 40X | 1/30 | 50 |  | Haze reduction | 250 | 156 | 95 |

**
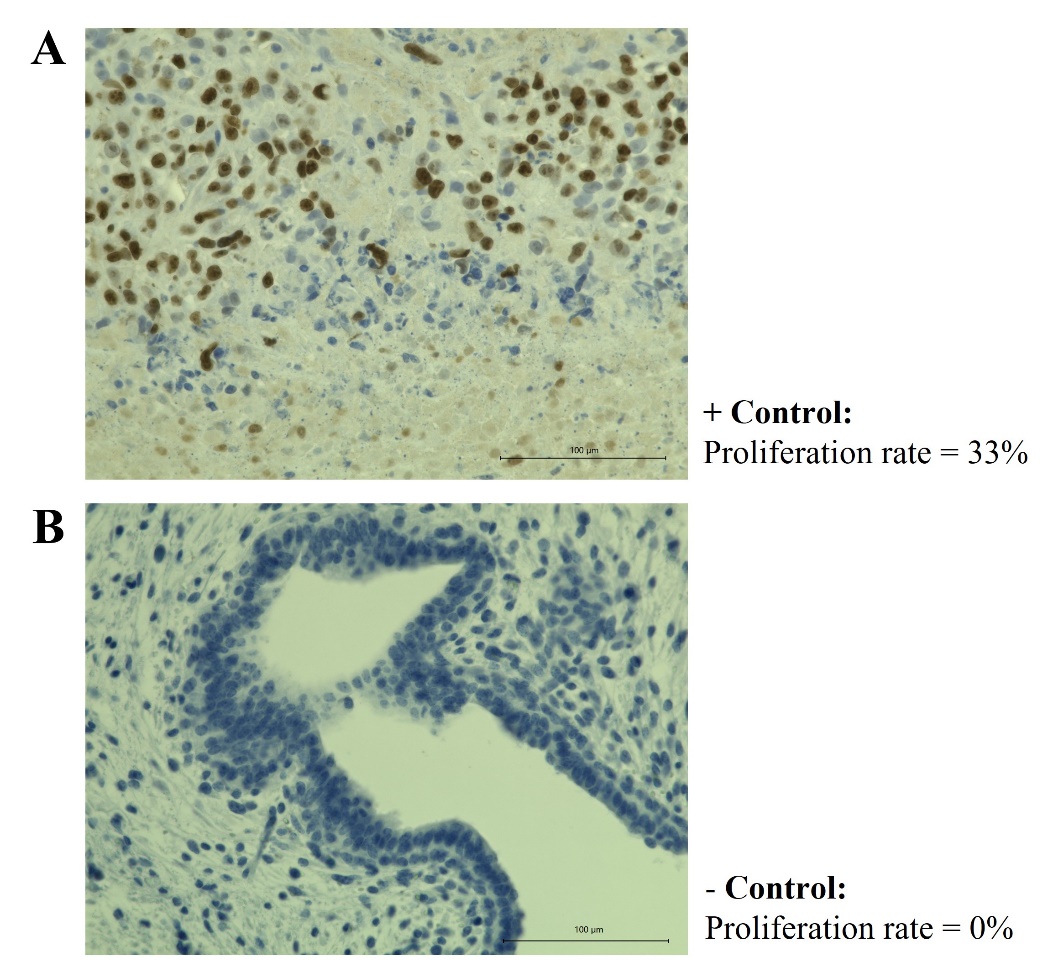
**

**Supplementary Figure S1.** Photomicrographs of calf jejunum (positive-Ki67 control, **A**) and mammary parenchyma with no primary antibody incubation (negative-Ki67 control, **B**). Images were captured at 40X (scale bar = 100 μm) and Ki67-positive cells were detected using the BZ-X800 Bioanalyzer.
